# More ‘milk’ than ‘psychology or tablets’: Mental health professionals’ perspectives on the value of peer support workers

**DOI:** 10.1111/hex.13151

**Published:** 2020-12-12

**Authors:** Timothy Moore, Laetitia Zeeman

**Affiliations:** ^1^ Sussex Partnership NHS Foundation Trust Horsham UK; ^2^ School of Health Sciences University of Brighton Brighton UK

**Keywords:** discourse analysis, experiential knowledge, lived experience, mental health, mental health professionals, peer support, psychology, psychosocial, subjectivity, user involvement

## Abstract

**Background:**

Though growing numbers of peer support workers are employed in the UK National Health Service (NHS), conflicts persist between core values of peer support and values which exert power within these services.

**Objectives:**

To explore what NHS mental health professionals value about the peer support worker role.

**Design:**

Five professionals from different professions and mental health settings were interviewed twice. The first interviews explored their experiences of working with peers. Transcripts were analysed using discourse analysis and psychosocial theory. Second interviews allowed participants to respond to the analysis and influence subsequent analysis.

**Results:**

Mental health professionals valued peers for the deeply empathic, relational approach they brought, based in their subjective experience. Peer work was also valued for the affect‐focused quality of this work, and the challenge peers pose to existing values in mental health services. The values of peer support troubled dominant ways of working based in forms of knowledge that favour objectivity and hence encountered challenges.

**Conclusions:**

Peers fulfil the role of amplifying the status of diverse forms of knowledge, values and related ways of working that have become marginalized in NHS mental health services. It is important that peers are not seen as an isolated solution to the marginalization of these forms of knowledge and values, but that their way of working becomes reflected in other roles whilst evoking change throughout these services.

**Patient or Public Contribution:**

Patient and Public Involvement groups were consulted both in the design and analysis stages of the study.

## 1 INTRODUCTION

Service user involvement has featured in UK National Health Service (NHS) mental health policy and planning for almost three decades. From the NHS and Community Care Act,^1^ through to the updated NHS Constitution^2^ and the Five Year Forward View for Mental Health Services,^3^ it seems clear that service user involvement is here to stay.

This paper presents a study of peer support as part of the service user/survivor involvement movement in mental health. Though definitions of peer support in contemporary mental health services vary considerably, all tend to coalesce around certain core features. Individuals offering peer support must have ‘lived experience’ of mental health problems (variously defined), which they use to support others through their own mental health problems. Beyond this, definitions differ in emphasis and interpretation. For example, most avoid diagnostic language, though some refer to ‘mental illness’,^4^ while others instead describe ‘challenges’^5^ or ‘distress’.^6^ Murphy and Higgins,^7^ and Watson^8^ give a finer grained description of this variability through their look at the values and critical ingredients of peer support, finding differences such as the degree of reciprocity in the relationship and the extent to which peer support is seen as a form of activism, seeking social change.

This definitional variability results from the diverse origins of peer support, most of which are grassroots and community‐based, grounded in practice and activist movements rather than top‐down guidance or policy.^8^ For health researchers, this variability is problematic because it renders the role and functions of peer support difficult to operationalize and measure.^7^ The worth of peer support is therefore not captured by the quantitative outcome research methodologies that top the post‐positivist ‘hierarchy of evidence’ and wield the most power in health‐care cultures.^9^, ^10^ Repper and Carter,^11^ in their review of outcome research, cite equivocal results associated with quantitative research and so turn to qualitative research to gain an understanding of the benefits of peer support. Watson^8^ points to quantitative studies providing evidence of slightly better outcomes for services which incorporate peer support, but again indicates that this kind of research is hampered by unclear definitions of role and function. Therefore, the evidence base to inform peer support is contested, emerging and tentative.

This variability can also be viewed as integral to the identity and philosophy of peer support. It is person‐centred and thus reflects the diversity of those it seeks to help, responding to a wide variety of identities, experiences, relations to and ways of managing distress in different historical and cultural contexts. In this, it differs from many other therapeutic approaches whose credibility within the NHS has required them to become more precisely defined. For example, though most psychological therapies originate in clinical practice,^12^ those prioritized in the NHS achieve this priority by building a research evidence base. The most influential forms of research evidence involve experimental designs requiring precise control over independent variables^9^; in this case, the therapy being offered. In order to be highly regarded, therapies must therefore be standardized (or ‘manualized’) so that statistically significant numbers of therapists can be considered to be delivering the same intervention. This restricts the person‐centredness of these therapies and leaves little room for therapists to use their personal experience as a therapeutic resource. Therapeutic practice must be similarly standardized so it can be claimed that what is being provided is in fact the same evidence‐based therapy.^13^ Peer support practice does not fit this model because it prioritizes the interaction of the individual subjective experience of the peer support worker with that of the service user and hence, as described, variability is integral. In this, it is importantly consistent with the values of the recovery movement, which defines individual service user's outcomes not in general terms imposed upon them by others (such as measurable symptom reduction), but instead insisting that recovery outcomes are defined by what holds meaning for the individual.^14^

Yet despite its lack of alignment with powerful discourses of evidence‐based practice in contemporary health care, there has been an expansion of peer support in the NHS.^8^, ^11^, ^15^, ^16^ This may be because other powerful agendas such as human rights^17^ and sustainability^18^ support the wider use of peer support in mental health services. Whatever the reasons, research indicates that the promotion of peer support in organizational cultures underpinned by values, models and systems of knowledge, which conflict with peer support's core values, has encountered problems.^19^, ^20^ In their review of evidence, Vandewalle et al^21^ describe how peer support workers often need to justify their role to colleagues and feel misunderstood and not valued. They also found difficulties of integration in teams lacking a recovery‐oriented culture and a lack of training and support. These factors acted as barriers to effective peer support where tasks and specialist techniques were prioritized over the formation of interpersonal relationships central to effective peer support. Given these tensions and barriers and the fact that professional roles are shaped by discourses which appear to conflict with core values of peer support, further research was needed to understand what it is about peer support that mental health professionals value. Whilst there are numerous studies investigating professional's perspectives on other forms of user involvement,^22^, ^23^, ^24^, ^25^, ^26^, ^27^, ^28^ few explore the views of other professional groups on peer support.^29^, ^30^

### 1.1 Objectives

This research explored the ways in which peer support workers are valued by mental health professionals. Frosh's psychosocial formulation of the subject as ‘a site, in which there are criss‐crossing lines of force, and out of which that precious feature of human existence, subjectivity, emerges’^31^ helps explain why the experience of mental health professionals is of interest. Those studies which explore professional's perspectives find that most claim to be pro‐peer support and user involvement,^29^, ^30^ but are simultaneously subject to forces exerted by models and cultures which conflict with its core values.^32^, ^33^, ^34^ Professionals’ subjectivity is therefore a site at which particular tensions interact and become manifest, and an examination of their experiences will shed light on these tensions. Frosh's formulation also resonates with the lead author's (TM) experience as a mental health professional and psychologist trying to implement various forms of user involvement and encountering resistance, both within himself in the form of anxiety and in his struggles to integrate this work with other imperatives associated with his professional role. The question therefore arose as follows: ‘what is it that mental health professionals value about peer support, given the tensions and difficulties it potentially arouses?’

## 2 METHODS

This paper presents part of the findings of an interview‐based study exploring resistances to user involvement initiatives within NHS mental health services. Interviews initially focused on describing what professionals valued and found meaningful, as this was necessary in order to describe and understand exactly what it was that was being resisted. This paper presents the findings from this initial section of the interviews as it yielded rich data.

Though participants were asked about all forms of service user involvement, they spoke most about peer support. There is arguably an important distinction between peer support and user involvement (in certain settings peer support could be provided by individuals who have not used services), but the findings reflect the participant's understanding of these terms.

### 2.1 Sampling and recruitment

Five practitioners were interviewed, purposively sampled to include as broad a range of professions and settings as possible (see Table 1). This was to ensure data represented a broad range of professional and organizational values and discourses. Given the detail of analysis required by the methodology used, which involved close reading of selected sections of text, five participants was considered an adequate number. All participants were employed in adult mental health settings based in the UK NHS and had experience of user involvement work within the previous six months. Participants were recruited via email from within one NHS Trust. The lead author had no working relationship with any of the participants.

**Table 1:** Participant details

Participant	Gender	Profession	Mental health work setting	Years post‐qualification experience
1	Female	Occupational Therapist	Acute Care	Over fifteen
2	Female	Psychologist	Community	Over fifteen
3	Female	Nurse	Community	Over fifteen
4	Male	Social Worker	Specialist Services	Over fifteen
5	Male	Psychiatrist	Community	Over fifteen

### 2.2 Data collection and analysis

Participants were interviewed twice. The first interviews lasted from forty‐five minutes to one hour and twenty minutes. Second interviews lasted from twenty‐five to forty‐five minutes. The gap between first and second interviews varied from three to five months. All participants chose to be interviewed at their workplace.

All interviews were conducted by the lead author. The first interviews explored participant's experiences of user involvement work, including when they had found it particularly valuable, and why. Interviewing was influenced by Hollway and Jefferson's^35^ Free Associative Narrative Interview approach, which promotes the development of narrative accounts through participants speaking about their experiences as freely as possible in order to allow the traces of discursive influences and the use to which they were put by participants to become evident. Interviews were semi‐structured; an interview schedule was used as a guide, but where participants were speaking freely, the schedule was used minimally. The schedule (see Appendix S1) was developed by the researcher in consultation with Patient and Public Involvement (PPI) groups (see section 2.4) and drawing on the lead author's skills as a psychological therapist.

Interviews were audio recorded, transcribed and analysed drawing on Willig's^36^ guidelines for Foucauldian Discourse Analysis, whilst utilizing Davies and Harré’s^37^ concept of positioning and psychosocial theory.^35^, ^38^ The lead author transcribed the interviews in order to retain as fully as possible the experience of the original interviews.^39^ Willig's guidelines were utilized to identify how discursive resources were used to construct the peer role, and the power and practices available to this role. Key discursive objects (eg expertise, risk, medical model, recovery model) were identified, and extracts which exemplified these discursive objects were selected for closer analysis. A psychosocial approach was used to look at how participants made use of the discursive resources identified to manage the anxieties associated with their work and experiences, to construct an acceptable sense of self within their work role,^35^ and their emotional attachment to and investment in particular discourses ^38^, ^40^, ^41^ This approach enabled an exploration of the interrelation of the subjective experience of participants for their practice (‘The little things’), the emotional impact of their experience (‘Embodied affect’) and the organizational context (‘Challenge’).

In the second interviews, the analyses of the first interviews were presented back to participants. They were asked to respond, given the opportunity to challenge or clarify interpretations and to add any further information. The second interviews increased rigour by providing a means of testing the meaningfulness and relevance of interpretations. ^35^, ^40^ The second interviews were audio recorded but not transcribed. They were used to inform the on‐going analysis of data from the first interviews, as described in the Results section. Patient and Public Involvement (PPI) groups were similarly used in the analytic process (see section 2.4).

### 2.3 Reflexivity

Reflexive note taking was used extensively throughout the research process. Berger's^42^ three‐part log approach was used to structure these notes, focussing on the interviewer's initial interpretations of data and on his subjective emotional responses. These emotional responses were used to guide the selection of text for closer analysis. Psychosocial researchers^38^, ^40^, ^41^ drawing on psychoanalytic practices, stress the importance of the careful and judicious use of the researcher's subjective responses. As Frosh and Saville‐Young^40^ assert, analyses developed in this way should be tested by sharing them with the original participants. Therefore, participants were interviewed twice. This testing was further strengthened by additionally presenting the initial interpretive analyses to PPI groups. The responses of participants at second interview and the PPI groups also informed which extracts were selected for final analysis, these being the extracts which held most meaning and emotional resonance.

The lead author is a Counselling Psychologist working in the NHS. He has significant experience of implementing service user involvement initiatives, and of working alongside peer support workers.

### 2.4 Patient and public involvement (PPI)

Two PPI groups were consulted, using INVOLVE^43^ guidance. One group consisted of service users who were members of a forum informing mental health service developments, so had an interest in improving the quality of user involvement. The other was a multidisciplinary group of mental health professionals and hence corresponded to the participant group. Both groups were consulted during the planning and design of the project. As described above, both groups were consulted during analysis. Key extracts were presented, along with a draft analysis. The groups were invited to respond to both extracts and analyses, and these responses guided subsequent analysis.

### 2.5 Ethical Approvals

Ethical approval was obtained from the University Research Ethics Committee and through the Integrated Research Application System (IRAS Id: 237366).

## 3 RESULTS

The results are presented in three sections reflecting key aspects of the peer role and positioning within services, which emerged during analysis. The extracts were chosen using the process described above whereby initial selection was informed by the researcher's affective response and subsequent refinement of this selection was based on the responses of participants and PPI groups. This collaborative analytic process resulted in the selection of a small number of extracts for close analysis in the final stages; hence, only five extracts are presented here.

The first section describes the relational, empathic nature of peer's work, based in personal experience which enables identification with service users. The second looks at the embodied and non‐verbal quality of this work, and the third the nature of the challenge peers pose to existing values in mental health services.

### 3.1 The ‘Little’ Things

Extract one comes from a narrative about a peer worker voicing what they had felt important during their discharge from psychiatric hospital. Participant 1 (P1) describes how they voiced this whilst supporting a service user during the discharge process.

‘I remember once a…peer [support worker] saying, um, “And I was really worried who was gonna get the milk…to put in my fridge… um because, I didn’t feel up to going out…on the first day I was discharged home … but I didn’t like to say to anybody about ‘cos I thought they’d think I was silly.”’ (Extract 1. P1, Occupational Therapist. Lines 978‐81)

The narrative felt significant because it seemed an attempt to describe something difficult to express about the value of the peer worker; how they can voice things that others do not or cannot, yet which P1 said she considered ‘*really important’* (P1. Line 987). Milk seemed to be used to represent the kind of thing that peers are better at. So what is this?

P1 later describes it as ‘*the little … subtle things … that are really important to somebody’* (P1. Line 987). These things get lost amongst the different concerns of professionals:

‘as a professional it was kind of the last thing we’d necessarily’ve thought about…you’re so…busy about “Have they got the care plan? Have they got their discharge plan?”’ (Extract 2. P1, Occupational Therapist. Lines 982‐5)

A professional could have raised the issue of milk, but P1 says they do not because their role prioritizes care plans and discharge plans and marginalizes ‘little’ (yet ‘*really important*’) things like milk.

That professionals will prioritize care plans over milk is also indicated by the peer's concern that raising the same issue during their discharge would be perceived as ‘*silly*’. P1 presents the discharge process as a context in which specialized professional discourses carry weight whilst others, symbolized by milk, are less important, even silly. Voicing these other discourses as a service user, dramatically disempowered in this situation, was not possible. However, this individual's shift into the peer role enabled them to voice these discourses, and this is heard and valued by professionals such as P1. This shift from ‘*silly’* to ‘*really important*’ shows how the peer role elevates the validity of the discourses signified by milk so they can compete with professional discourses.

Because ‘milk’ felt so symbolically significant here, TM presented a draft analysis to participants and PPI groups for their opinion about what it symbolized. Their interpretations of the significance of milk extended the metaphor beyond what is evident in extract 1. They thought it a powerful symbol of ‘care and comfort’. They also reflected that in hospital milk is provided, so having to get your own milk at home would represent a loss of positive aspects of care available in hospital. If milk is ready for you at home, they said, this could feel like some of this care is carried over into the home and you are being held in mind at this vulnerable transitional point.

It is worth noting that this value is illustrated in part through contrasting it with what clinicians cannot do. The clinician is dominated by the powerful technical discourses which marginalize the care symbolized by milk. The prioritization of these technical discourses effectively limits the practices available to clinicians to tasks associated with these kinds of discourses, which another participant neatly summarized as ‘psychology or tablets’ (P4. Line 527).

Whilst P1’s symbol of milk is effective in illustrating a key value of the peer's role, her narrative also shows how this value is precarious. It is considered ‘really important’, yet P1’s description of the discharge process shows how professionals prioritize other tasks. The peer role could have been created in order that these things can be voiced, at least this is one reading of the value that P1 constructs here. The peer's value lies in their being able to voice things that the ‘lines of force’^31^ which operate upon professionals have marginalized to the point of exclusion.

### 3.2 Embodied affect

Participant two (P2) attempted to describe a unique quality in the relational work of peer support workers;

‘you could see the difference in, the, sort of the … quality of the connection…between…two peers, and, you know, a client and a therapist obviously, you know, that can be really helpful in…other ways but you could really see … the value in it.’ (Extract 3. P2, Psychologist. Lines 601‐4)

P2 here contrasts the value of the relationship between a peer and a service user with that developed with a therapist. She describes the latter as potentially ‘*really helpful’*, but the peer/service user connection has a valuable different ‘*quality*’. P2 twice refers to being able to ‘*see*’ this different quality, constructing it as visual, rather than verbal.

P2 had, earlier in the interview, made another attempt to describe the value of the peer's work:

‘the feedback we got from clients was that, you know it was so helpful to meet with someone who actually knew …. what it was like to receive that diagnosis, and to t‐…you know it’s that classic ‘They, they just get it’ you know ‘because they know it, they.ve been there, they know what it feels like to’ you know ‘ have those difficulties’ … umm and also I think they really appreciated that…because they knew that this person…knew where they were coming from…the peer support worker could probably be more frank with them…you know, so could … c‐ i‐it was a different they could have a almost a different kind of relationship’ (Extract 4. P2, Psychologist. Lines 216‐22)

In the notes made following this interview, TM noticed how P2’s first description (Extract 4), despite being verbally richer, made much less of an impact than the second (Extract 3). However, through the process of transcription, which reduces P2’s communication to written words, removing her tone of voice and visible presence, the impact of the second attempt was lost. In contrast to the detail given in extract 4, extract 3 simply appeals to a different quality of connection and being able to ‘*see*’ the value. So why did this description feel so much more powerful?

Just as P2 described the quality of the connection between peer and service user as visual, the researcher's reflective notes described having been able to *see* a different quality in her communication when she described the value the second time, such that this description had a more powerful emotional impact. This different quality was visual (in her posture) and audible (in her tone), both non‐verbal qualities that are lost in transcription.

Hollway^39^ writes about the difficulty of retaining the vitality of meanings conveyed during interviews using conventional social science methods, which strip out meaning that cannot then be recaptured. Transcription loses non‐verbal aspects of communication such as rhythm, pace and emphasis. Holloway^39^ promotes the use of observational methods, drawn from infant observation in psychoanalytic training, which offer ways of capturing embodied affect, including the use of the researcher's subjective emotional responses. The impact of P2’s description on TM depended upon non‐verbal aspects which had to be witnessed. Without these, the impact is lost. Because the impact of her communication is largely non‐verbal, this parallels the point she is making about the embodied, non‐verbal nature of the value of the peer's work.

At the second interview, the analysis was fed back to P2 and she agreed with his interpretation of what was illustrated. She said that the interaction between peer worker and service user must be witnessed, as opposed to read about, to truly appreciate its value. She suggested this could be problematic if managers influencing practice lack such direct experience, because this means they cannot properly value peer work or appreciate what they are trying to achieve, thereby risking tokenism.

In the light of what this section shows about the non‐verbal, embodied nature of the peer's work, P1’s choice of milk to symbolize the value of the peer's work appears more apt. As already described, milk expresses care, comfort, empathy and thoughtfulness, but it also expresses physicality, embodiment and not only the non‐verbal, but the preverbal. Milk is central to the bodily exchanges of early maternal care. These exchanges are physical and preverbal, they shape early identity/personality formation,^44^ whilst providing the primary ingredient for physical, bodily growth and require the (close) physical presence of both mother and infant. Milk thus becomes suggestive of fundamental interpersonal processes, and the importance of physical presence in these processes.

### 3.3 Challenge

This extract was selected because, as a mental health professional himself, the researcher empathized with the sense of embarrassment described by this participant. These feelings seemed to indicate a point of tension and it was hoped that closer analysis would clarify what was being shown in this emotive communication.

Participant three (P3) described challenging existing practice as a valuable function of the peer's role:

‘the peer worker was in the room and one of the doctors said something like…”Well I haven’t got time to ask about people’s carers” … and I was just really conscious that she was in the room…and, I mean i‐i‐it was a bad thing to say anyway d’you know what I mean, but it wa‐ it just seemed much much worse, it really just shone the light on the ….”Really!?”’ (Extract 5. P3, Nurse. Lines 415‐9)

P3 clearly felt critical of the doctor's comment, but the peer's presence magnified her feelings such that she cringed when describing this. There was a tension between discourses represented by the doctor (the biomedical model which locates mental health problems and treatment within the body) and the peer (a social model which stresses the importance of supportive relationships), exacerbated by time constraints which forced prioritization of one discourse over another.

For P3, the presence of the peer amplified the challenge to the dominant biomedical discourse. Importantly, the impact here was again non‐verbal. The peer worker did not speak, their simple physical presence evoked an uncomfortable emotional response, perhaps shame, in P3. Such a response occurs through a process of identification. P3 spontaneously imagined what the psychiatrist's utterance sounded like from the peer's point of view, and this amplified the feeling of wanting to challenge the doctor that was already in her. This was a non‐verbal process grounded in shared values and similarity.

It is also important to note the ambivalence and tension here. Though P3 clearly valued the challenge, she experienced being challenged as uncomfortable. This may have been because she was part of the same organization as the doctor and so probably also identified to some extent with their position. Whilst the challenge was welcomed, the associated emotional experience might not have been. If such emotions are not reflected upon and understood they could be avoided by, for example, excluding the peer from such consultations. This would be an example of unexamined affect leading to resistance to meaningful integration of peers.

## 4 DISCUSSION

Perhaps the most useful definition emerging from the study data is that what professionals value about the peer support role is the ability to offer ‘milk’, contrasted with the ‘psychology or tablets’ prioritized by professionals, positioned as they are by the ‘lines of force’^31^ which operate within mental health service cultures and which produce their roles and subjective experience.

‘Milk’ symbolizes the practice of peers; the care they provide by using their lived experience to relate empathically. It is important to note that certain psychotherapeutic traditions identify a similar kind of care as fundamental to therapy. Humanistic and person‐centred therapies place empathy and a relational focus at the centre of the therapeutic endeavour.^45^, ^46^ In psychodynamic and psychoanalytic therapies the therapist's subjective responses (countertransference) fundamentally inform the therapeutic process.^47^ In these therapies the status of subjective, experiential knowledge and non‐verbal interpersonal communication is elevated in relation to theoretical knowledge and technique.^48^, ^49^

But these therapeutic approaches tend to be marginalized within the NHS. This marginalization is arguably a product of characteristics shared with peer support, such as the focus on relationship and allowing the client to direct the change process, which mean both peer support and these therapies develop in ways that cannot be standardized and their outcomes are not predefined. This puts both in tension with the powerful discourses of evidence‐based practice, described in the introduction.

This study illustrates how forces operate to marginalize the very features of peer support that are ostensibly valued. This was evident in ‘The Little Things’ where powerful specialist discourses, represented by P1 as care plans and discharge plans, were prioritized over ‘milk’, and indeed could make it seem ‘little’ and ‘silly’. This finding resonates with assertions that any form of user involvement will struggle in cultures which privilege specialized ways of talking and forms of knowledge which are objective and unemotional, medical or managerial^33^, ^34^ and undermine or exclude others.

Hollway^41^ suggests that the dominance of these forms of knowledge in psychological research serves a defensive function. She describes how the objectification of those researched, required by dominant methodologies, produces difference and distance between researchers and those researched. She uses Kleinian psychoanalytic theory to illustrate how characteristics deemed bad or unacceptable are split off and projected into those being researched, allowing researchers to retain valued characteristics such as agency and reason. This unconscious process produces difference and, given its emotionally defensive function, will resist change. A similar process can occur between mental health professionals and service users. Menzies‐Lyth^50^ identified how institutional defences operate to impose emotional distance between general nurses and those they care for. This distancing protects against the emotional impact and intimacy of the nursing role, but is also detrimental to the quality of care. Others^51^, ^52^, ^53^, ^54^ describe similar processes in the field of mental health whereby qualities such as vulnerability, fragmentation, unreason or divergence from societal norms are projected into mental health service users. Through producing distance, these processes are likely to reduce empathy, affect quality of care and impede recovery.

The peer support worker role can be seen as an attempt to break down the differences upon which this defensive distancing is based by providing a bridge between professional and service user. Hollway^41^ makes a plea for basing psychological research in processes which focus on shared subjective experience, because such sharing enriches the subjectivities of both researcher and researched. The peer worker's careful, reflective use of their subjectivity and identification with service users represents a similar move within mental health services. Though this use of subjectivity and empathy is valued by professionals, the approach is likely to struggle to find space in cultures where knowledge grounded in objective, distancing epistemologies dominates.

## 5 CONCLUSION AND PRACTICE IMPLICATIONS

This paper describes how peer support workers are valued by other mental health professionals. This value lies in a quality of relational, empathic, embodied care which they provide, in their use of and the value their role attaches to subjective, experiential knowledge and in the challenge that the promotion of this care and this knowledge represents to ingrained cultures within statutory mental health services. But these qualities and characteristics are not unique to peer support workers. They are, in different ways and to different degrees, exhibited by other professionals who draw on life‐experiences (which may include experience of mental health problems) and subjective experiences within therapeutic relationships. Professionals can work relationally and empathically and can challenge dominant discourses in mental health services, even if the psychosocial ‘lines of force’ acting upon them put them at risk of experiencing conflicts similar to those described by peers.^20^, ^21^

Watson^8^, ^20^ describes how political influences and organizational contexts shape the peer role, the practices available to it and the subjective experience of it. She cites the distress of peers struggling to maintain cherished values in contexts where incompatible demands are made of them.^20^ It is vitally important that these functions and practices, so valued by peers and other mental health professionals, are not exclusively located in peers. This would both impoverish professionals, rendering them less able to use their subjectivity, and construct the peer role as a simplistic solution to what is a complex and entrenched problem of cultural change in mental health services. Such change requires the engagement of all members of that culture, and attending to intrapsychic processes as well as interpersonal interactions and organizational processes. Peers are a valuable vanguard for both the provision of ‘milk’ and raising its status in relation to the currently more highly valued ‘psychology or tablets’. However, we propose that sharing of practice, learning and experience with other mental health practitioners is vital. Peer workers can learn from the experience of therapeutic traditions that use subjectivity, and professionals must learn about the peer worker's experiential approach and values and respond thoughtfully and collaboratively to the challenge they bring. There must be shared training, supervisory and reflective spaces in which this sharing can occur so that peer support, whilst maintaining and developing its identity, can be an integrated part of the social change which it was originally intended to catalyse.^20^

### 5.1 Limitations and strengths

Though attempts were made to recruit professionals from a range of settings and different professions, the work context of the participants remains specific. All worked within one mental health Trust in the NHS. The experiences of those working in different context will differ, and hence, the themes and issues identified here cannot be generalized. In addition, the recruitment strategy purposively selected professionals who were open to speak about user involvement and peer support. It is likely that this resulted in the over‐representation of practitioners in favour of user involvement. A further study would benefit from more reflexivity around recruitment, including asking participants why they decided to take part.

The second interview provided a means of testing analytic interpretations by checking the degree to which relevant groups recognized them and found them meaningful.^35^, ^40^ However, further meetings with participants would have produced richer data. Much psychosocial research involves multiple interviews, which enable a deeper reflexive dialogue to develop.^55^, ^56^ This would have allowed greater exploration and elaboration of the most relevant and meaningful discursive aspects as they emerged and provided a way of both testing and extending the interpretations produced here regarding the complexities surrounding peer support, the hopes it may carry for clinicians, the ambivalent feelings they may have about it and how all of these are produced by the social context of the work. A greater appreciation of these complexities will be necessary for the future success of peer support in the NHS.

## CONFLICTS OF INTEREST

Both authors confirm no conflicts of interest.

## DISCLAIMER

This paper represents independent research funded by the NIHR. The views expressed are those of the authors and not necessarily those of the NHS, the NIHR or the Department of Health and Social Care.

## Supporting information

Appendix S1

## Data Availability

The data that support the findings of this study are available from the corresponding author upon reasonable request.
