# Supplementary material for: More ‘milk’ than ‘psychology or tablets’: Mental health professionals’ perspectives on the value of peer support workers
Source: Health Expect. 2020 Dec 12;24(2):234–42. doi: 10.1111/hex.13151 (PMC8077122; doi:10.1111/hex.13151)
Supplement: Supplementary file 1 — Appendix S1 [file HEX-24-234-s001.docx]

## **APPENDIX: Interview Schedule**

This schedule shows the first two questions from a longer schedule which also asked about problems encountered in implementing service user involvement. Only the first two questions are included here as they represent the section of the interviews focusing on the perceived value of user involvement and peer support.

| **Interview Prompt Sheet**  *Primary aim is to hear in detail about experiences and associated feeling. Prompts are to be used only if necessary to help participants describe their experiences.*  …………………………………………………………………………………………………………………….  **1** - Can you describe your first experience of SUI work?  PROMPTS: *Setting? Purpose/function? Types of people involved? What was it like starting up? How did you feel about it? How did it develop/change over time?*  Have you had other experiences since? Describe them too. (*List instances to use as a prompt in subsequent questions*)  **1a** - Is this work important to you? Why/What do you feel is important about it?  PROMPTS: *What is it about you that makes you interested in it? When did it become so? Which of the experiences you described worked best? Why?*    **2** - What has been really meaningful/successful, as opposed to ‘tokenistic’ SUI? (*discuss what term they would like to use here*) *(seek descriptive examples)*  **2a** - How did you know it was meaningful/successful/*own term*? Tell me about what made you realise this.  **2b** - What were the meaningful/successful/*own term* results you saw? Can you describe how they became apparent?  **2c** - What do you think aids/contributes towards/enables this success? Can you give me an example of how you have seen this work? |
| --- |
